# DUSP15 expression is reduced in the hippocampus of *Myrf* knock-out mice but attention and object recognition memory remain intact

**DOI:** 10.1371/journal.pone.0281264

**Published:** 2023-02-02

**Authors:** Florence Rawlings-Mortimer, L. Sophie Gullino, Sebastian Rühling, Anna Ashton, Chris Barkus, Heidi Johansen-Berg

**Affiliations:** 1 Wellcome Centre for Integrative Neuroimaging, Nuffield Dept of Clinical Neurosciences, Oxford, United Kingdom; 2 Department of Pharmacology, University of Oxford, Oxford, United Kingdom; 3 Department of Neuroradiology, School of Medicine, Technical University of Munich, Munich, Germany; 4 Nuffield Department of Clinical Neurosciences, Sleep and Circadian Neuroscience Institute, University of Oxford, Oxford, United Kingdom; 5 Department of Experimental Psychology, University of Oxford, Oxford, United Kingdom; Nathan S Kline Institute, UNITED STATES

## Abstract

The atypical protein tyrosine phosphatase enzyme, dual-specificity phosphate 15 (DUSP15) is thought to be activated by myelin regulatory factor (MyRF) and to have a role in oligodendrocyte differentiation. Here, we assess whether *Dusp15* is reduced in the hippocampus of mice with conditional knock-out of *Myrf* in oligodendrocyte precursor cells. Using quantitative polymerase chain reaction (qPCR) we found that Dusp15 expression was indeed lower in these mice. Alterations in myelin have been associated with Alzheimer’s disease (AD), autism spectrum disorder (ASD) and attention deficit/hyperactivity disorder (ADHD). Symptoms of these disorders can include impairments of object recognition and attention. We, therefore tested the mice in the object recognition task (ORT) and 5-choice serial reaction time task (5CSRTT). However, we did not find behavioural impairments indicating that attentional abilities and object recognition are not impacted by reduced oligodendrogenesis and hippocampal *Dusp15* expression. Gaining insight into the role of newly formed oligodendrocytes and *Dusp15* expression is helpful for the development of well targeted treatments for myelin dysregulation.

## Introduction

Oligodendrocytes produce myelin in the central nervous system (CNS). Oligodendrocytes develop from oligodendrocyte precursor cells (OPCs) which express platelet-derived growth factor receptor alpha (PDGRFα). Myelin wraps around the axons of neurons to facilitate neurotransmission. Alterations in CNS myelination have been reported in a wide number of disorders including Alzheimer’s Disease (AD), Multiple sclerosis (MS), bipolar disorder and schizophrenia [1, 2]. Myelin regulatory factor (MyRF) [3] is a membrane-associated transcription factor that directly binds myelin gene enhancer regions and is important for myelin formation. MyRF has also been implicated in driving remyelination [4]. To investigate the role of MyRF in behaviour in the adult mouse, MyRF^(-/-)^ mice that have selective deletion of *Myrf* in PDGRFα positive cells and exhibited loss of newly formed mature oligodendrocytes were used [5]. MyRF^(-/-)^ mice have been shown to have impairments in some behaviours including learning complex wheel running [5] spatial memory [6] and contextual Pavlovian conditioning [7]. However, whether other behaviours including attention and short-term object recognition memory are affected by disrupted oligodendrogenesis has not been investigated.

Some effects of MyRF on myelination and remyelination may be mediated by its downstream targets. Dual-specificity phosphate 15 (DUSP15) is a protein tyrosine phosphatase enzyme with ability to dephosphorylate phospho-tyrosine and phospho-serine/threonine residues [8]. DUSP15 is thought to be activated by both MyRF and Sox 10 [9] and is present in both Schwann cells [10] and oligodendrocytes [11]. DUSP15 was found to be required for extracellular-regulated protein kinase 1 and 2 activation in Schwann cells [10]. Other identified substrates of DUSP15 include platelet derived growth factor beta (PDGRFβ) and sorting nexin 6 (SNX6) [11]. There are conflicting reports on the role of DUSP15 in oligodendrocyte differentiation. Previous literature found that silencing *Dusp15* in-vitro resulted in oligodendrocyte differentiation, suggesting that DUSP15 may be a negative regulator of this process [11]. Nonetheless, another study found that DUSP15 was upregulated in differentiating oligodendrocytes and reduced DUSP15 levels lead to corresponding decreases in markers of differentiation such as Nkx2.2 and Mag [9].

Given that DUSP15 appears to be activated by both MyRF and Sox10, understanding whether *Myrf* conditional knock-out (cKO) results in altered expression of *Dusp15* and whether cognitive processes are impacted could prove useful in the development of well targeted therapeutics. Many diseases exhibit alterations in myelination such as Multiple sclerosis (MS), Alzheimer’s Disease (AD) and childhood autism spectrum disorder (ASD). Object recognition and attentional impairments are common in MS and AD [1, 12, 13]. DUSP15 has also been associated with ASD [14, 15] which often co-occurs with attention deficit/hyperactivity disorder (ADHD) [16, 17]. Altered white matter has been widely associated with ADHD for meta-analysis see [18, 19]. Changes in white matter microstructure including decreased fractional anisotropy (FA) and increased radial diffusivity (RA) were found in children with ADHD suggesting altered myelination [20]. Corpus callosum size was also found to correlate negatively with inattention and hyperactivity in older male adults [21]. Previous research has linked altered myelination with impaired object recognition. For example, mice that lack glycoprotein CD133 have reduced numbers of OPCs and mature oligodendrocytes along with short-term object recognition impairment [22]. Object recognition was also impaired in mice that had focal ischemic lesions of the corpus callosum [23].

We hypothesised that *Dusp15* expression would be reduced in MyRF^(-/-)^ mice. We found that this was the case with lower *Dusp15* expression observed in the hippocampus of MyRF^(-/-)^ mice compared with their MyRF^(+/-)^ sibling controls. We also hypothesised that MyRF^(-/-)^ mice would have an impairment in short-term object recognition memory and that reduced attention may explain these impairments. We therefore tested the mice in the object recognition task (ORT) and 5-choice serial reaction time task (5CSRTT). However, we did not find any difference in the performance of the MyRF^(-/-)^ mice, indicating that oligodendrogenesis and *Dusp15* expression in the hippocampus are not essential for these behaviours.

## Methods

### Experimental mice

All experiments were undertaken at the University of Oxford under UK home office project licence (P78348294) and approved by the Animals (Scientific Procedures) Act 1986. Mice with floxed myrf around exon 8 [24] were crossed with PDGRFα-CreERT2 mice [25] and Rosa26R-eYFP reporter mice [26]. These MyRF^(-/-)^ mice therefore had selective deletion of myrf in platelet-derived growth factor receptor α positive cells when administered tamoxifen. Hemizygous littermates MyRF^(+/-)^ were used as controls. MyRF^(-/-)^ and MyRF^(+/-)^ mice were housed in groups of two to five under a 12-hour light/dark cycle. Behavioural training and testing were performed during the light phase at the same time each day.

### Tamoxifen administration

Male and female MyRF^(-/-)^ and MyRF^(+/-)^ mice were administered tamoxifen (Sigma, 300mg/kg) at postnatal day 70 for four consecutive days via oral gavage. The tamoxifen solution was prepared freshly on each day of administration by dissolving in corn oil (Sigma) to a concentration of 40mg/mL. The mice were given at least three weeks to recover from any side effects of the tamoxifen, such as weight loss, prior to behavioural testing.

### Behavioural testing

#### Object recognition task

The mice were trained and tested in a 26cm square arena. All sessions were recorded with an overhead camera and the amount of time the mice spent exploring the objects was scored manually offline. Exploration was counted if a mouse showed interest in the object by sniffing, touching or climbing on it. The objects used (plastic unicorn toy, wooden pegs and highlighter pen) were of similar height (~8cm) but varied in texture and shape. The mice were able to rear up and climb on the objects that were secured to the floor of the arena. The box and objects were cleaned with Anistel (1:200) between sessions.

*Habituation*. The mice initially underwent a 4min habituation session without objects present. Twenty-four hours later they underwent a second 4min habituation session with a highlighter pen secured in the centre of the arena for them to explore.

*Training*. Twenty-four hours after the second habituation session, mice were trained in the same arena with two identical objects either unicorn toys or pegs, counterbalanced between groups, for 4mins.

*Testing*. Two hours after the training session the mice were placed back into the arena for 4mins with one of the old objects they encountered during the training session and a new object (either the unicorn toy or pegs).

#### 5-choice serial reaction time task

Mice were trained in four operant boxes (Med Associates, Albans, VT) placed inside a sound attenuating chamber. On one wall of the operant box there was a panel of five nose poke holes with recessed LED stimulus lights. The opposite wall contained a food reward port where strawberry milkshake (Yazoo kids) was dispensed following correct responses. For a correct response the mouse needed to nose poke into the hole that had been illuminated within a specific time, either whilst the light was on or for the limited hold (LH) period that followed. This resulted in strawberry milkshake being available at the reward port. Nose poking into a non-illuminated hole was classed as an incorrect response. Five second time out periods, in which the house light was switched on and the nose poke holes were unresponsive, were given if a mouse made an incorrect response, failed to respond within the LH or responded prematurely during the inter-trial-interval (ITI). Performance was assessed using several different measures including percentage choice accuracy, percentage of correct responses, percentage of omitted responses, percentage of premature responses and percentage of perseverative responses.

*Food restriction*. The mice were food restricted to around 85–90% of their starting body weight four days prior to the start of habituation and maintained at this weight throughout the training and testing periods.

*Habituation*. The mice were initially habituated to the strawberry milkshake used as the reward in their home cages on two occasions, once before the start of food restriction and once after it had been initiated. The mice were then habituated to the operant boxes for 4–6 days. On the habituation days the mice underwent one 30-min session where all of the nose poke holes were illuminated until nose poked, with an ITI of 2 seconds (sec). Nose pokes resulted in strawberry milkshake being available at the reward port. Once the mice had learnt this association and nose poked at least 50 times in one session, they were able to progress to training.

*Training*. There were seven stages in the training period during which the difficulty of the parameters was gradually increased. The mice underwent one session per day consisting of 100 trials. The sessions lasted for a maximum of 30-min or ended once 100 trials had been completed. In the first stage the stimulus duration (SD) was 20sec, the LH was 30sec and the ITI was 2sec. To meet criteria and move on to the next stage the mice needed to make 30 or more correct trials and get 30 percent correct for two consecutive days. In the final training stage, the SD was set at 0.8sec, the LH was 7sec and the ITI was 5sec, with a criterion of 50 correct trials, at least 80 percent accuracy and less than 40 percent omissions. For a full list of the parameters used during the intervening stages see [27, 28] S1 File.

*Stabilisation*. Once the mice had successfully completed training stage seven, they undertook at least five further days at these same baseline parameters (SD = 0.8sec, LH = 7sec and ITI = 5sec) before testing commenced.

*Testing*. Following the stabilisation period, the mice underwent a testing period which included four different tests A-D, undertaken on separated days and interspersed with two baseline days (averaged for the statistical analysis). The baseline days enabled the performance of the mice to recover to pre-test performance levels at baseline parameters so that the effect of each test could be reliably measured. In test A, the SD was reduced from 0.8sec to 0.4sec with all other parameters kept the same as baseline. In test B, the ITI was increased from 5sec to 7sec. In test C the SD was reduced from 0.8sec to 0.3sec. In test D white noise burst occurred at one of four time points, 0.5, 2.5, 4.5 and 5 seconds prior to stimulus onset. In one in every five trials, no white noise distractor was presented. Trial type was selected from a list of these five options without replacement, so one of each would occur within a set of five trials and a maximum of two of the same type of trial could occur sequentially.

### Immunohistochemistry

After behavioural testing the mice were euthanised using pentobarbital and perfused with 4% paraformaldehyde (PFA, Sigma-Aldrich). The brains were kept in PFA for 24hrs and then placed in 20% sucrose prior to embedding. The tissue was cryosectioned at ~ 30μm thick coronal sections and stored in 50% glycerol at -20°C prior to free-floating immunohistochemistry. For the immunohistochemistry the brain sections were initially washed in PBS, followed by 0.05M Tris-buffered saline (TBS, Sigma-Aldrich) under slight agitation at room temperature (RT) for 20min. After washing the sections were blocked with 10% foetal bovine serum (FBS, Thermo Fisher Scientific) and 0.5% Triton X-100 (Sigma-Aldrich) in TBS at RT for 2 hours. They were then incubated with primary antibodies anti-green fluorescent protein (anti-GFP 1:750, Alves Labs) and anti-adenomatous polyposis coli clone CC-1 (CC1 1:200, Calbiochem) in 5% FBS and 0.25% Triton X-100 in TBS at 4°C for ~16hours. Secondary antibodies, goat anti-chicken 488 (1:500, A11039, Thermo Fisher Scientific) and goat anti-mouse Alexa Flour 568 (1:500, A21144, Thermo Fisher Scientific) in 1% FBS and 0.1% Triton X-100 in TBS were applied at RT for 1.5hours. The nuclei were stained using Hoechst (1:1000, 33342, Thermo Fisher Scientific) in PBS at RT for 5min. The sections were mounted with fluorescent mounting medium (Dako) and stored at 4°C prior to microscopy. Confocal microscopy was undertaken using a FV1000 microscope (Olympus) with Fluoview software. For each animal three coronal sections were imaged at 20X magnification. Z-stack images (10 steps, 1.3μm step interval) were taken of each hemisphere from the midline of the corpus callosum to the corner of the lateral ventricle. A total of six images were counted per animal. The maximum intensity of the z-stacks was analysed using Fiji software (imagej.net/Fiji). A region of interest of 553μm x 169μm was defined in the corpus callosum and the number of YFP^+^/CC1^+^ cells were counted manually using the cell counter plugin.

### Quantitative PCR

A subset of mice were culled using rising CO_2_, their brains quickly removed and placed in ice-cold PBS. The brains were then placed into an ice-cold rodent brain matrix, sliced coronally, and tissue punches taken from the hippocampus using a biopsy punch with plunger tool (Miltex). Tissue samples were snap frozen on dry ice and stored at -80°C until RNA extraction. Total RNA was extracted using a RNeasy Plus Mini kit (Qiagen) according to manufacturer’s instructions. RNA was quantified using a NanoDrop spectrophotometer (Thermo Scientific) and RNA reversed transcribed to cDNA using a qScript cDNA synthesis kit (Quanta Biosciences). mRNA expression was determined by qPCR using primers against *Dusp15* and reference genes, *Gusb*, *Actb* (Table 1), using a QuantiFast SYBR Green PCR kit (Qiagen) on a StepOnePlus Real-Time PCR System (Applied Biosystems). mRNA expression was determined using the relative standard curve method, and *Dusp15* expression was normalised to reference gene expression.

**Table 1:** Sequences of primers used for qPCR.

	Forward primer (5’-3’)	Reverse primer (5’-3’)
***Dusp15***	CCTGGACTCTACCTTGGAAAC	GTGGATAAAGTGGACGCATTC
***Gusb***	CCAGAGCGAGTATGGAGCAG	TCGTCATGAAGTCGGCGAAA
***Actb***	CCACACCCGCCACCAGTTCG	TACAGCCCGGGGAGCATCGT

### Statistical analysis

Two-way mixed ANOVA, independent t-test or Mann-Whitney U tests were used. For the ORT the exploration time and object discrimination index were calculated as outlined in [29]. For the 5CSRTT the percentage accuracy, correct, premature, omissions, response latency and reward latency were calculated as outlined in [28] S1 File.

Two-way mixed ANOVAs were used in the 5CSRTT to test for differences between the cKO and hemizygous groups and within subject measures of timepoint (Baseline days vs test day). Data were analysed with R studio (version 2021.09.2). Data are presented as mean ± standard error of the mean (SEM); graphs were generated in GraphPad Prism (version 9.3.0).

## Results

### Reduced numbers of newly formed oligodendrocytes in MyRF^(-/-)^ mice

**Fig 1:**
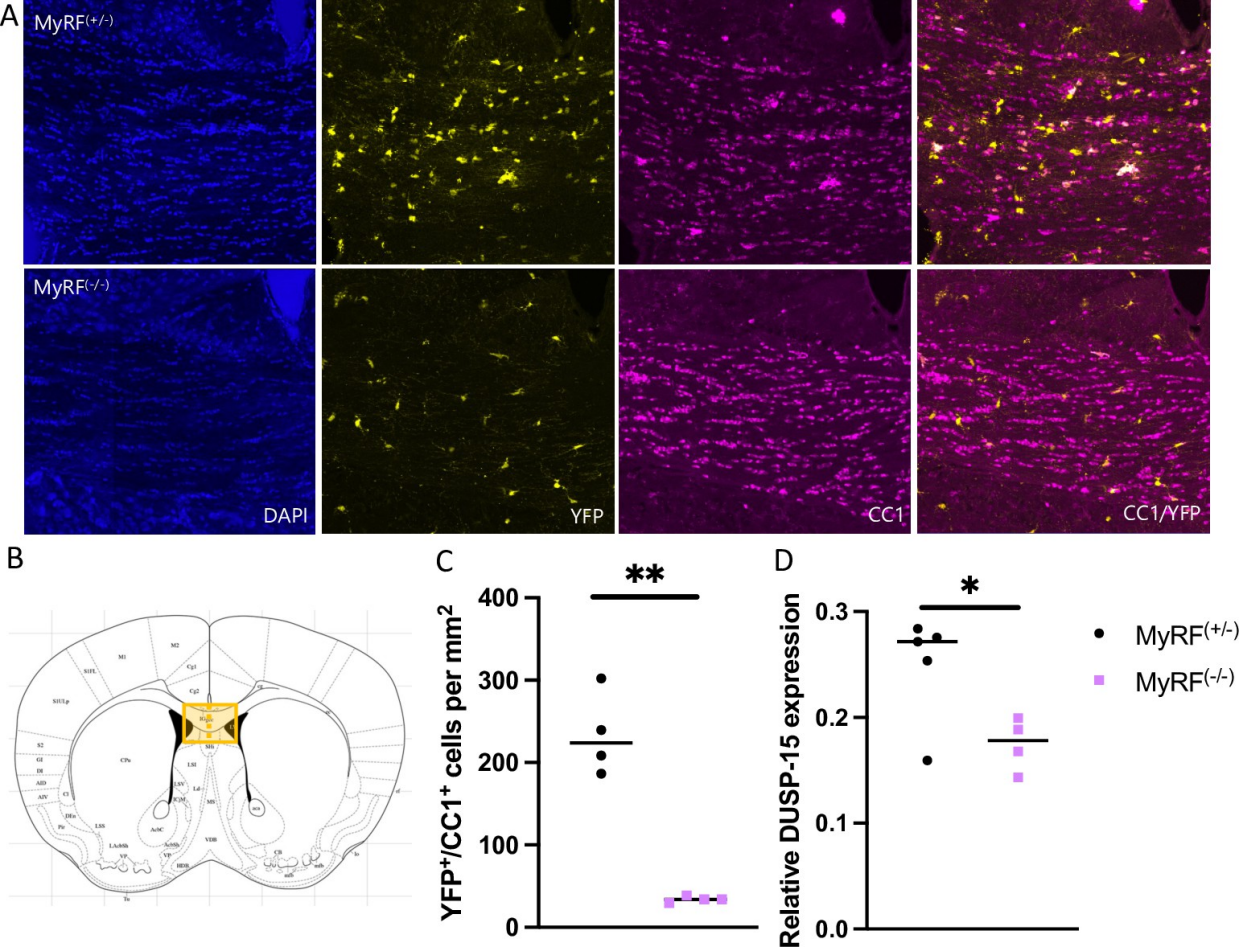
Reduced numbers of newly formed oligodendrocytes and *Dusp15* expression in MyRF^(-/-)^ mice. A) Representative histology images from MyRF^(-/-)^ and MyRF^(+/-)^ mice. B) Diagram showing representative region of the corpus callosum stained (bregma 0.86mm; interaural 4.66 according to the Paxinos and Franklin mouse brain atlas). For each animal three coronal sections were imaged with two images counted per section C) The number of YFP^+^/CC1^+^ cells was reduced in the corpus callosum of MyRF^(-/-)^ mice. D) *Dusp15* expression was found to be lower in the hippocampus of MyRF^(-/-)^ mice. Data presented mean ± SEM **p = 0.004, *p = 0.029.

The number of CC1^+^/YFP^+^ cells was found to be lower in the MyRF^(-/-)^ mice (n = 4, m = 1/f = 3) compared with MyRF^(+/-)^ (n = 4, m = 1/f = 3) controls (t = 7.94, DF = 3.03, p = 0.004, Fig 1C). CC1 is a marker of mature oligodendrocytes therefore this finding indicates successful knock-down of newly formed mature oligodendrocytes in the MyRF^(-/-)^ mice.

### Reduced *Dusp15* expression in the hippocampus of MyRF^(-/-)^ mice

*Dusp15* expression was found to be lower in the hippocampus of MyRF^(-/-)^ mice (n = 4, m = 2/f = 2) mice compared with MyRF^(+/-)^ controls (n = 5, m = 3/f = 2; t = 2.85, DF = 5.99, p = 0.029, Fig 1D).

### Object recognition memory intact in MyRF^(-/-)^ mice

**Fig 2:**
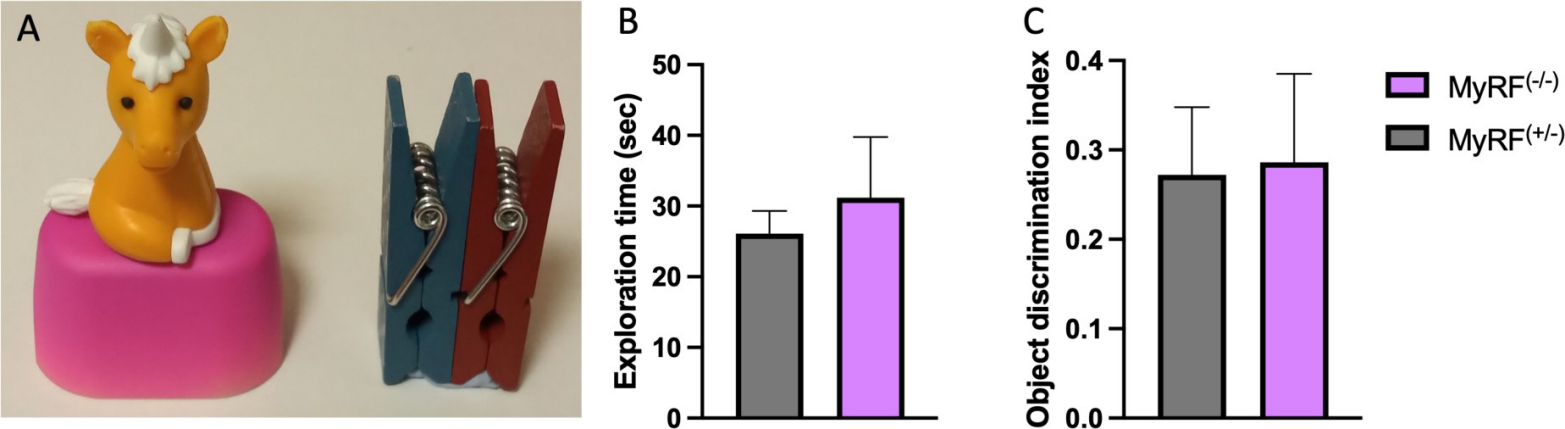
Object recognition memory in MyRF^(-/-)^ and MyRF^(+/-)^ mice. A) Objects used for object recognition testing. B) No difference seen in exploration time during the training session or C) object discrimination index during testing. Data presented mean ± SEM.

A total of 24 mice were tested in the ORT. The MyRF^(-/-^ (n = 12, m = 8/f = 4) and MyRF^(+/-)^ (n = 12, m = 6/f = 6) mice spent a similar amount of time exploring the objects during the training stage (w = 73, p = 0.977, Fig 2B). The object discrimination index was also similar for both the MyRF^(-/-)^ and MyRF^(+/-)^ mice (t = 0.116, DF = 20.57, p = 0.908, Fig 2C) during the test session. This revealed that both groups spent a similar amount of time exploring the new object compared with the old during the test session, indicating intact object recognition memory.

### Behaviour in the 5CSRTT intact in MyRF^(-/-)^ mice

**Fig 3:**
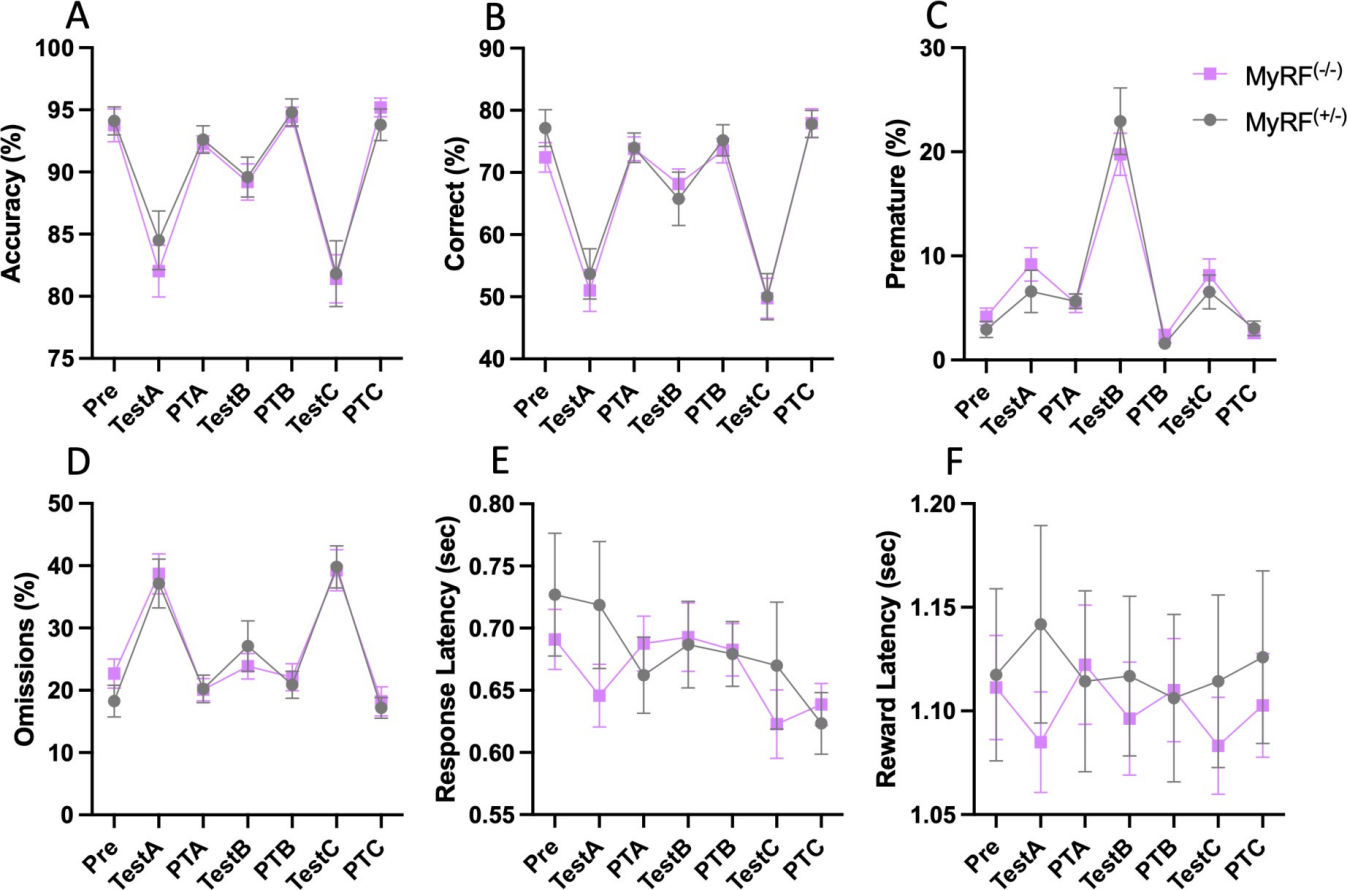
MyRF^(-/-)^ and MyRF^(+/-)^ have equivalent behaviour in the 5-choice serial reaction time task. No differences were seen in A) accuracy B) correct choices C) premature responses D) omissions, E) response latency F) or reward latency during test A-C. Data presented mean ± SEM. Abbreviations: day before testing started (Pre), post-test A (PTA), post-test B (PTB), post-test C (PTC).

A total of 49 mice were tested in the 5CSRTT. Five mice (2x MyRF^(-/-)^ and 3x MyRF^(+/-)^) did not reach the criteria level for testing and were excluded from the analysis. 38 mice (22x MyRF^(-/-)^ and 16x MyRF^(+/-)^) undertook tests A-C, 44 mice (26x MyRF^(-/-)^ and 18x MyRF^(+/-)^) undertook test D. Both groups successfully completed training with an average of twenty-eight training days undertaken prior to the start of testing (w = 224, p = 0.429). There were no differences seen between groups during tests (A-C). Two-way mixed ANOVAs were used to test for differences between the MyRF^(-/-)^ and MyRF^(+/-)^ mice in choice accuracy (Fig 3A), correct responses (Fig 3B) omissions (Fig 3C), premature responses (Fig 3D), response latency (Fig 3E) and reward latency (Fig 3F) for tests A-C.

For test A in which the SD was reduced from 0.8sec to 0.4sec, no main effects of genotype were seen in choice accuracy (F_1,36_ = 0.391, p = 0.536), correct responses (F_1,36_ = 0.515, p = 0.478), omissions (F_1,36_ = 0.354, p = 0.555), premature responses (F_1,36_ = 0.763, p = 0.388), response latency (F_1,36_ = 0.564, p = 0.458) or reward latency (F_1,36_ = 0.174, p = 0.679). Likewise, there was no timepoint x genotype interaction for choice accuracy (F_1,36_ = 0.535, p = 0.588), correct responses (F_1,36_ = 0.843, p = 0.434), percentage of omissions (F_1,36_ = 0.939, p = 0.396), premature responses (F_1,36_ = 1.133, p = 0.328), response latency (F_1,36_ = 2.170, p = 0.122), or reward latency (F_1,36_ = 2.613, p = 0.08). An effect of test was found for percentage accuracy (F_1,36_ = 50.76, p<0.001) and percentage correct (F_1,36_ = 110.08, p<0.001) with a decrease seen from baseline. This would be expected when the SD is reduced indicating that the mice were performing as expected. There was also an increase in the percentage of omissions (F_1,36_ = 75.04, p<0.001) and premature responses (F_1,36_ = 12.277, p<0.001) again this would be expected with reduced SD.

For test B in which the ITI was increased from 5sec to 7sec, no main effects of genotype were seen in choice accuracy (F_1,36_ = 0.108, p = 0.744), correct responses (F_1,36_ = 0.004, p = 0.953), omissions (F_1,36_ = 0.064, p = 0.802), premature responses (F_1,36_ = 0.384, p = 0.54), response latency (F_1,36_ = 0.131, p = 0.719) or reward latency (F_1,36_ = 0.004, p = 0.947). Likewise, there was no timepoint x genotype interaction for choice accuracy (F_1,36_ = 0, p = 1), correct responses (F_1,36_ = 0.703, p = 0.499), percentage of omissions (F_1,36_ = 1.123, p = 0.331), premature responses (F_1,36_ = 0.986, p = 0.378), response latency (F_1,36_ = 0.365, p = 0.696), or reward latency (F_1,36_ = 1.152, p = 0.322). There was however an effect of test for percentage accuracy (F_1,36_ = 15.72, p<0.001) and percentage correct (F_1,36_ = 10.72, p<0.001) showing a decrease from base line which would be expected when the ITI is increased. The percentage of omissions (F_1,36_ = 5.887, p = 0.004) and premature responses (F_1,36_ = 100.596, p<0.001) increased, again this is expected when the ITI is increased.

As we did not see any effect of reducing the SD to 0.4sec in test A, we therefore reduced it further in test C to 0.3sec to test if genotype differences would be present at an extremely stringent SD. However again we found no main effects of genotype on choice accuracy (F_1,36_ = 0.0.17, p = 0.898), correct responses (F_1,36_ = 0.035, p = 0.852), omissions (F_1,36_ = 0.034, p = 0.855), premature responses (F_1,36_ = 0.373, p = 0.545), response latency (F_1,36_ = 0.087, p = 0.77) or reward latency (F_1,36_ = 0.176, p = 0.677). Likewise, there was no timepoint x genotype interaction for choice accuracy (F_1,36_ = 0.371, p = 0.692), correct responses (F_1,36_ = 0.144, p = 0.866), percentage of omissions (F_1,36_ = 0.164, p = 0.849), premature responses (F_1,36_ = 0.603, p = 0.55), response latency (F_1,36_ = 1.485, p = 0.234), or reward latency (F_1,36_ = 0.690, p = 0.505). There was again a decrease from base line for percentage accuracy (F_1,36_ = 79.529, p<0.001) and percentage correct (F_1,36_ = 149.25, p<0.001), which is expected when the SD is reduced. The percentage of omissions (F_1,36_ = 96.987, p<0.001) and percentage of premature responses (F_1,36_ = 21.863, p<0.001) were also increased again.

**Fig 4:**
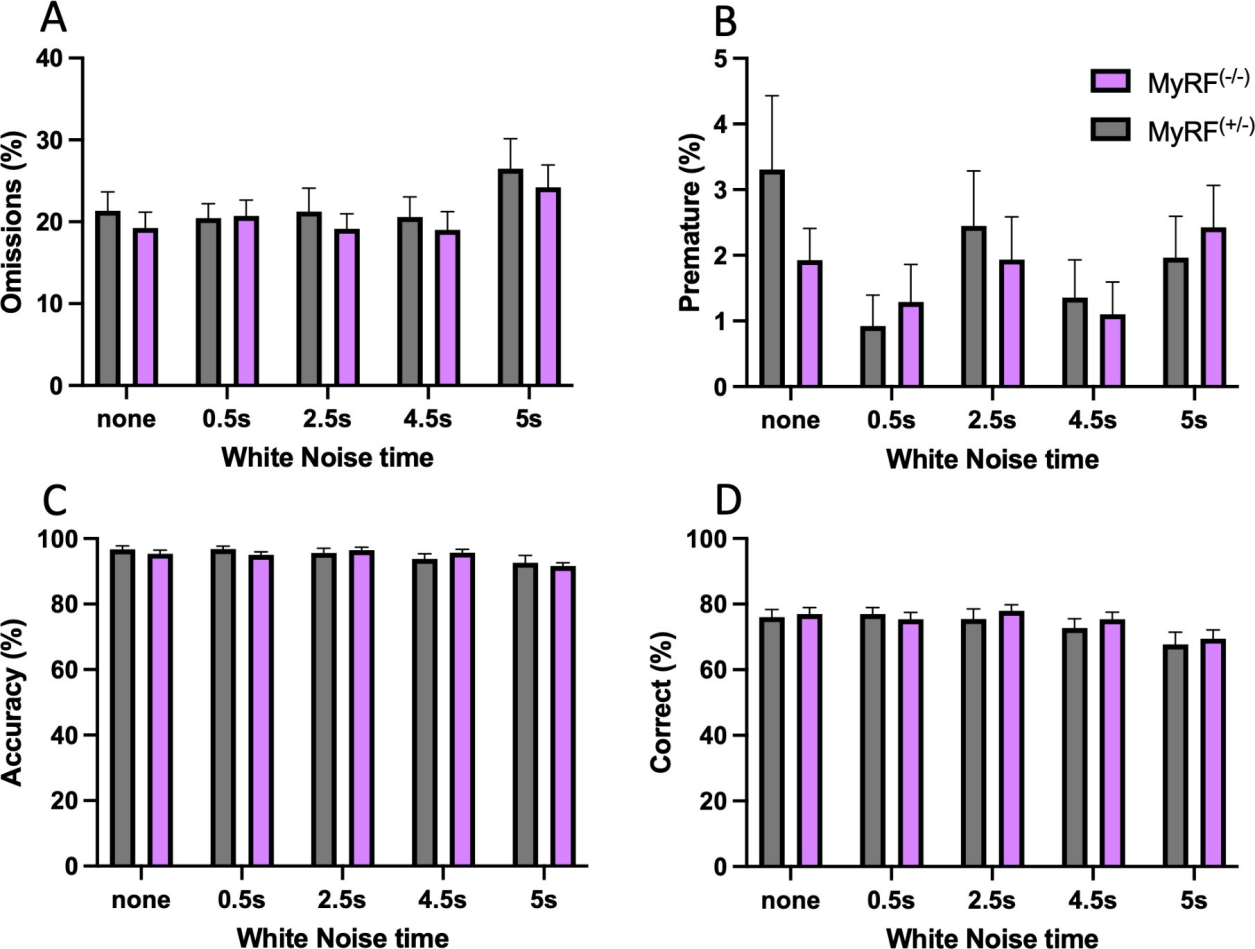
White noise bursts did not impact 5CSRTT performance in the MyRF^(-/-)^ mice compared with MyRF^(+/-)^ controls. No differences were seen in A) omissions B) premature responses C) accuracy or D) correct responses. A white noise burst was presented at one of four time points, 0.5, 2.5, 4.5 or 5 seconds prior to stimulus onset. In one in every five trials, no white noise distractor was presented. Data presented mean ± SEM.

MyRF^(-/-)^ (n = 26, m = 13/f = 13) and MyRF^(+/-)^ (n = 18, m = 10/f = 8) mice were tested with white noise distraction bursts at various timepoints during the session (test D; Fig 4). Again, there were no differences between the two groups with no main effect of genotype seen for choice accuracy (F_1,42_ = 0.059, p = 0.81), correct responses (F_1,42_ = 0.198, p = 0.659), omissions (F_1,42_ = 0.335, p = 0.566), or premature responses (F_1,42_ = 0.265, p = 0.61). There was also no genotype x white noise burst timepoint interaction for choice accuracy (F_1,42_ = 1.230, p = 0.299), correct responses (F_1,42_ = 0.633, p = 0.639), omissions (F_1,42_ = 0.223, p = 0.925) or premature responses (F_1,42_ = 0.782, p = 0.538).

## Discussion

We hypothesised that conditional deletion of Myrf would lead to reduced expression of *Dusp15* and would be associated with behavioural impairments in attention and short-term memory. MyRF^(-/-)^ mice were found to have reduced numbers of CC1/YFP positive cells in the corpus callosum indicating that the tamoxifen treatment protocol resulted in successful recombination and loss of newly formed oligodendrocytes in these mice. We found that *Dusp15* expression was lower in the hippocampus of these mice when compared with their hemizygous siblings. This finding agrees with previous research indicating that *Dusp15* is activated by MyRF [9]. However, in contrast to our hypothesis, we did not find evidence for impairments in short-term object recognition memory, or in attention of MyRF^(-/-)^ mice, indicating that these processes remain intact despite the reduction in hippocampal *Dusp15* and oligodendrogenesis.

Our data suggests that hippocampal DUSP15 isn’t involved in short-term object recognition memory and attention. Previous literature on the role of DUSP15 in behaviour is limited. One study found that expression decreased during morphine-induced conditioned place preference (CPP), whereas overexpression in the nucleus accumbens (NAc) promoted extinction of CPP [30]. *Dusp15* overexpression, however, did not improve impaired short-term spatial memory following repeated morphine exposure in the Morris watermaze [30]. Another study found that when *Dusp15* was overexpressed, there was a transient increase in myelin basic protein (MBP) followed by a decreased after around six days indicating that DUSP15 may have inhibiting effects on myelination [9]. In-vitro knockout of *Dusp15* was also found to increase expression of myelin markers including Mbp, myelin-associated glycoprotein and connexin32 [10]. It is possible that the reduced levels of *Dusp15* we found in the hippocampus of MyRF^(-/-)^ mice resulted in the upregulation of myelin by pre-existing oligodendrocytes which could potentially have compensated for the loss of newly formed oligodendrocytes. Recent research has indicated that pre-existing oligodendrocytes can undergo myelin reorganisation following skilled reaching training [31, 32]. Although, it should be noted that newly formed MBP^+^ cells have been shown to be reduced in the medial prefrontal cortex of MyRF^(-/-)^ mice [24].

Previous research found that MyRF^(-/-)^ mice had impaired complex wheel learning [5] and short-term spatial memory [6] indicating that new oligodendrocytes are required early in the learning and memory process [33]. Therefore, we expected that MyRF^(-/-)^ mice would have similar impairments with short-term recognition memory and that an inability to pay attention may explain this impairment. However, we did not find impairments in either the short-term ORT or the 5CSRTT considered here. These findings suggest that MyRF^(-/-)^ mice have intact short-term recognition memory, suggesting that new oligodendrocytes are not required for this process. This finding fits with other research that did not find differences in short-term Pavlovian association memory, instead seeing differences thirty days after conditioning [7]. We chose to focus on short-term ORT memory in this study. However, it is possible that long-term ORT memory is impaired in these mice. Future work could explore this possibility. The MyRF^(-/-)^ mice could also have impaired social memory. A number of tasks could be used to assess for this including the three-chamber social approach or two-trial social memory test [34–37].

In conclusion we found that *Dusp15* expression is lower in the hippocampus of MyRF^(-/-)^ mice. Gaining a detailed understanding of the downstream targets of MyRF is important as it could help with treatment of demyelinating diseases such as MS. It is also useful to gain further insight into the types of behaviour that are affected by reduction of newly formed oligodendrocytes. We did not find evidence for impairments in short-term object recognition and sustained attention and impulsivity in MyRF^(-/-)^ mice, suggesting that reduced oligodendrogenesis does not cause behavioural deficits consistently across all cognitive domains.

## Supporting information

S1 File (DOCX)
