# Supplementary material for: DUSP15 expression is reduced in the hippocampus of Myrf knock-out mice but attention and object recognition memory remain intact
Source: PLoS One. 2023 Feb 2;18(2):e0281264. doi: 10.1371/journal.pone.0281264 (PMC9894471; doi:10.1371/journal.pone.0281264)
Supplement: S1 File — (DOCX) [file pone.0281264.s001.docx]

Data available at https://doi.org/10.6084/m9.figshare.21940220.v1.
